# Retraction: Redox Regulation of the AMP-Activated Protein Kinase

**DOI:** 10.1371/journal.pone.0355536

**Published:** 2026-08-12

**Authors:** The PLOS One Editors

The *PLOS One* Editors retract this article [1] due to concerns about reliance on retracted work cited in References 26, 27, 28, 29, 30, 31, 34, and 35.

All authors either did not respond directly or could not be reached.

Additionally, concerns were noted that Fig 3A AMPK lanes 2–5 appear similar to the Fig 4C AMPK panel, despite being used to represent different experimental conditions. In light of the retraction of this article [1], PLOS has not investigated these concerns further.
